# The Method of Minimal Residual Disease Detection With Circulating Tumor DNA and Its Clinical Applications in Colorectal Cancer

**DOI:** 10.1002/cnr2.70167

**Published:** 2025-03-04

**Authors:** Ming Liu, Tianhao Mu, Jia Gu, Mingyan Xu, Shifu Chen

**Affiliations:** ^1^ HaploX Biotechnology Shenzhen China; ^2^ Faculty of Data Science City University of Macau Macau China

**Keywords:** circulating tumor DNA, clinical applications, clinical trials, colorectal cancer, minimal residual disease

## Abstract

**Background:**

Colorectal cancer (CRC) remains a significant health concern in the world. The existing standard of care guidelines for CRC surveillance fall short of effectively and timely detecting recurrence or metastasis.

**Recent Findings:**

In recent years, circulating tumor DNA (ctDNA) has emerged as a promising material for minimal residual disease (MRD) detection. In this article, we provide an exhaustive review of the methods utilized for MRD detection via ctDNA, present evidence supporting the potential of ctDNA MRD as a valuable biomarker in clinical applications, and engage in a discussion regarding ongoing ctDNA MRD‐based clinical trials in CRC. Finally, we offer insights into future prospects of ctDNA‐based MRD methodological advancements and clinical research.

**Conclusion:**

It is foreseeable that more sensitive, flexible, and economical MRD detection methods will emerge with the deeper research on cell‐free DNA (cfDNA) genomics, fragmentomics, methylomes, and nucleosome imprinting. At the same time, MRD‐guided intervention studies will evolve for revolutionizing the treatment paradigm of CRC.

## 1 Introduction

Colorectal cancer (CRC) stands as the third most common cancer worldwide and the second leading cause of cancer‐related deaths. It was projected that over 1.9 million new CRC cases and nearly 1 million deaths occurred globally in 2020 [1]. The prognosis varies based on the stage of CRC. Early‐stage CRC patients have a higher chance of cure and a better prognosis, with the five‐year survival rate for localized CRC around 90%. Advanced or metastatic CRC patients, however, have a lower survival rate, with a five‐year survival rate of approximately 14% [2]. The current standard of care guidelines for CRC surveillance primarily rely on radiographic and tumor biomarker evidence. However, this approach requires improvements in accuracy and sensitivity [3]. Tumors can be small or present subtle changes, making them challenging to detect or differentiate accurately from normal tissue. Moreover, early detection of micro‐metastases is crucial for effective treatment and improved patient outcomes, but they may not be detectable by conventional imaging methods even when they have spread to distant sites.

Minimal residual disease (MRD) refers to a phenomenon where a small number of tumor cells or their derivatives remain in the body during the clinical remission period post‐treatment. These residual cells often show no signs or symptoms and may not be detectable by traditional imaging or protein methods. However, they can be the root cause of tumor recurrence. Presently, liquid biopsy is emerging as a new method to detect MRD. Solid tumors can release certain tumor biomarkers into body fluids, which include circulating tumor cells, circulating tumor DNA (ctDNA), circulating tumor RNA, proteins, extracellular vesicles, and tumor‐educated platelets [4]. In addition to blood, urine, peritoneal fluid, and feces are also useful materials for liquid biopsy [5].

The detection and characterization of ctDNA are the most commonly used and extensively researched methods for MRD detection. ctDNA, first reported in 1977 [6], refers to the DNA fragments derived from cancer cells present in body fluids, which are part of cell‐free DNA (cfDNA). ctDNA can be detected based on the identification of various tumor‐specific genetic or epigenetic aberrations, such as single nucleotide variations (SNVs), insertions and deletions (indels), amplifications, translocations, chromosomal aberrations, methylation, and fragmentomic changes [4]. Studies are increasingly demonstrating that ctDNA is an ideal material for MRD detection [7]. With the improvement of ctDNA detection methods, very low levels of tumor genomic or epigenetic alterations in ctDNA can be detected, demonstrating high sensitivity and specificity in detecting MRD. Furthermore, ctDNA analysis is a non‐invasive and convenient method that requires only blood or other body fluid samples from the patient. Importantly, ctDNA provides real‐time information about the tumor burden or treatment response, which is useful in making personalized treatment decisions. Numerous clinical guidelines and expert consensus have affirmed the application value of ctDNA testing in monitoring postoperative recurrence risk, predicting therapeutic efficacy, and assisting therapeutic decision‐making [8, 9, 10]. This can help patients achieve comprehensive management from the early to the late stages of tumors. The discussion section of the 2021 v2 version of the National Comprehensive Cancer Network (NCCN) Guidelines for CRC has explicitly stated that ctDNA MRD testing can assess the risk of CRC recurrence, but it requires further clinical research for validation [9]. In essence, it recognizes the value of MRD in assessing the risk of postoperative recurrence of CRC, but more clinical research is still needed to guide adjuvant chemotherapy (ACT).

Although MRD detection with ctDNA is still an evolving field and further research is needed to optimize its clinical utility and establish standardized protocols for its implementation, it is a promising tool in the management of CRC. This review will discuss the methods for MRD detection with ctDNA, the evidence supporting ctDNA MRD as a promising biomarker in current clinical research, and the ongoing ctDNA MRD‐based clinical trials in CRC.

## 2 ctDNA‐Based MRD Detection Methods

**FIGURE 1:**
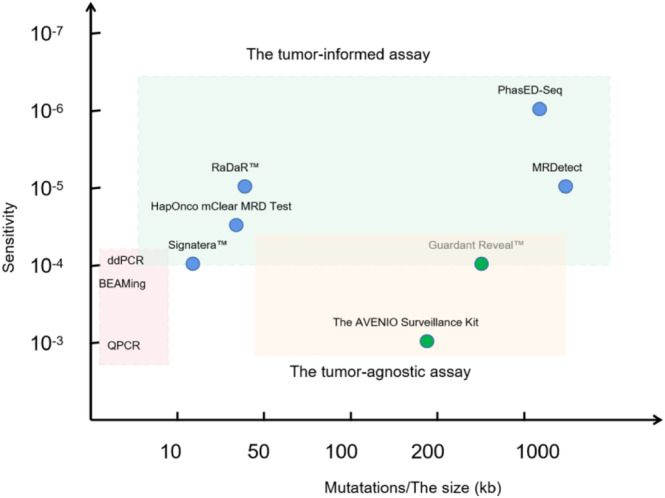
Approaches for ctDNA‐based MRD testing and sensitivity considerations. For PCR or personalized ctDNA‐based MRD assay, the number of mutations targeted and the approximate sensitivity for ctDNA that can be achieved are shown. For tumor‐agnostic ctDNA‐based MRD assay, the size of fixed panel and the approximate sensitivity for ctDNA that can be achieved are shown. The classic representatives of commercial kits are also shown. Blue circles represent the personalized tumor‐informed assay. Green circles represent the tumor‐agnostic assay. BEAMing, “beads, emulsions, amplification, and magnetics”; ddPCR, digital droplet PCR; kb, kilobase pair. QPCR, quantitative PCR.

ctDNA‐based MRD detection in CRC presents several challenges. First, the tumors in CRC patients exhibit heterogeneity in genomic profiling [11], and each patient may carry only a few of the same gene mutations, making it challenging to design a universal panel. Additionally, sensitivity is a significant bottleneck. ctDNA level is a crucial representation for MRD detection, but the amount of ctDNA released into peripheral blood is generally very small. TracerX studies show the linearity of tumor size and blood ctDNA concentration, with each cubic centimeter of tumor lesion equivalent to 0.19 copies of the tumor genome per milliliter of plasma, implying that ctDNA abundance is about 0.01%–0.02% [12]. Therefore, the sensitivity of ctDNA detection needs to reach the order of 1/10,000 when performing MRD detection. Finally, technical errors and biological background noises derived from clonal hematopoiesis are inevitable in ctDNA detection [13], which can introduce false positives without effective elimination methods. A variety of approaches for ctDNA‐based MRD testing have been developed currently (Figure 1).

The most direct method for detecting ctDNA is through polymerase chain reaction (PCR). The limit of detection of PCR is significantly enhanced by digital droplet PCR and a related technique known as BEAMing (beads, emulsions, amplification, and magnetics). These techniques can boost sensitivity by 10–100 times compared with conventional PCR methods. Research has demonstrated their ability to consistently detect variant allele frequencies (VAFs) ranging from 0.1% to 0.01% [14]. However, these technologies are not without their limitations. Their capacity to target only individual or a minimal number of predefined mutations restricts their flexibility and scalability, especially in MRD settings where simultaneous tracking of multiple mutations is often necessary for ctDNA detection.

To overcome these challenges, innovative approaches based on next‐generation sequencing (NGS) have been developed. Additionally, several comprehensive strategies have also been introduced. First, multiple genomic or epigenetic alterations are tracked concurrently, thereby increasing the probability of ctDNA detection [15]. Unique Molecular Identifier methods are incorporated into the NGS pipeline to minimize PCR and sequencing artifacts [16]. Furthermore, paired peripheral blood mononuclear cells are sequenced alongside cfDNA to filter out false positive variants originating from clonal hematopoiesis [17]. Finally, ctDNA MRD detection methods can be classified into tumor‐informed and tumor‐agnostic categories, depending on prior knowledge of the tumor mutation profile. Typically, tumor‐informed methods focus on known tumor mutations. In contrast, tumor‐agnostic approaches do not require prior knowledge of the tumor mutation profile and can also include DNA methylation profiling.

Tumor‐informed methodologies utilize the mutation profile derived from the primary tumor tissue, focusing specifically on these known mutations for ctDNA MRD detection. These methods significantly enhance the sensitivity of MRD detection by conducting deep sequencing in targeted genomic regions. Concurrently, they are effective in minimizing background noise generated from non–tumor‐derived mutations. Generally, tumor‐informed techniques employ targeted NGS sequencing, which encompasses strategies such as multiplex PCR or hybrid capture [18]. Signatera (Natera Inc., San Carlos, CA, USA) is a notable example of this approach. This assay commences with whole exome sequencing of the tumor tissue, and the top 16 somatic mutations are selected for corresponding cfDNA detection. Primers for amplifying these targets are then designed, and 16‐plex PCR capture followed by ultra‐deep sequencing up to 100 000× is performed on cfDNA. cfDNA that detects at least two targets is classified as MRD positive. Remarkably, this assay has a detection limit of 0.01% VAF [19]. Several studies have verified the clinical utility of this MRD detection assay in CRC [20, 21, 22]. RaDaR is another tumor‐informed method designed to track 48 tumor‐informed variants for MRD detection, boasting a VAF detection limit of 0.001% [23]. MRDetect is a tumor‐informed assay based on whole genome sequencing (WGS) for the detection of MRD. It applies WGS to tumor tissue to provide information for each personalized ctDNA assay. Each somatic SNV identified in the tumor is then examined in cfDNA to calculate the cumulative whole genome tumor signal from plasma samples. The copy number aberrations (CNAs) obtained through tumor sequencing are similarly checked in plasma cfDNA, and combined with SNV data to generate a statistical detection score. Notably, MRDetect demonstrates high sensitivity in ctDNA detection, even in cases where the tumor fraction is as low as 0.001% and the sequencing depth is 35× [24]. PhasED‐Seq is a tumor profiling sequencing method based on hybrid capture. It utilizes the detection of phase variations, that is, two or more mutations occurring within a 150 base pair region on the same DNA strand, to achieve high sensitivity in ctDNA detection. Notably, the performance of PhasED‐Seq surpasses that of dual‐strand sequencing based on SNVs, with a detection limit lower than one in a million [25].

Tumor‐agnostic methods eliminate the need for personalized tumor sequencing. Given the inherent limitations of a fixed gene panel approach, these assays often incorporate epigenomics, including aberrant methylation [26]. Guardant Reveal is a prime example of this approach. It employs a fixed 500‐kilobase gene panel to execute hybrid capture‐based NGS. By amalgamating genomic and epigenomic signatures, it demonstrates the ability to detect ctDNA in early‐stage CRC patients with 91% sensitivity, down to a detection limit of 0.01% VAF. Furthermore, it can be performed serially at various intervals to enhance sensitivity. It exhibited impressive performance in detecting MRD in a prospective observational study involving a 103‐patient cohort at stages I–IV CRC undergoing curative intent therapy [27]. However, in the COBRA study, a prospective phase II/III clinical trial evaluating ctDNA‐guided ACT in resected stage IIA colon cancer, patients who had detectable ctDNA after surgical resection showed no improvement in ctDNA clearance following 6 months of chemotherapy compared to patients who were only monitored, which is not consistent with other reports [28, 29]. The specificity of this assay is questionable [30]. The AVENIO ctDNA Surveillance Kit is a specialized NGS liquid biopsy assay designed to help researchers monitor tumor burden in lung and CRC over time and to assess for MRD. This assay encompasses 471 mutation‐prone regions associated with disease presence across 197 genes, including those listed in the NCCN Guidelines. This panel demonstrates > 99% specificity and > 99% positive predictive value for all classes of mutations, with a detection limit of 0.1% VAF at the time of diagnosis [31], while evaluating its applicability and performance for MRD detection should be addressed in future studies.

Both technical schools boast unique strengths. The primary advantage of tumor‐informed assays lies in their ability to customize the number of panel tests based on a patient's specific tumor, thereby avoiding wasteful sequencing due to individual differences. Moreover, they offer exceptionally high sensitivity. The drawback is that they necessitate tumor tissues and cannot detect new mutations that arise during treatment. Additionally, their turnaround time is longer, making them most suitable for scenarios demanding very high sensitivity, such as post‐curative surgery. On the other hand, tumor‐naive assays bring the benefit of using fixed panels, eliminating the need for customization for each patient. They do not depend on tumor tissue and can detect new mutations occurring during treatment. However, these assays require more data, possess relatively lower sensitivity, and there is a potential for false negatives. They are most apt for patients with advanced tumors due to the high tumor loads and extremely high levels of ctDNA these patients present. Furthermore, new drug‐resistant gene mutations might surface during the treatment of late‐stage patients, which necessitates evaluation through a large panel [32]. HaploX has innovatively developed the HapOnco mClear MRD Test, which employs a strategy combining 40 customized loci and a 21 fixed gene panel. This approach effectively merges the advantages of both tumor‐informed and tumor‐naive assays. Besides, it is important to note that the cost of sequencing assays, excluding the methylation PCR‐based tests, can be prohibitive, particularly in healthcare systems where resources are limited. This is indeed a significant challenge that affects the accessibility and implementation of personalized treatments based on tumor profiling.

In recent years, it is noteworthy that ctDNA methylation alone has demonstrated significant potential as a biomarker for MRD detection. Unlike the first generation of Guardant Reveal, where methylation was merely a key component, the latest version of the Guardant Reveal assay can detect the methylation status of over 2000 differentially methylated regions. The sensitivity and specificity of this latest generation have reached impressive levels of 81% and 98.2%, respectively [33]. Cai and his team devised a sophisticated multiplexed quantitative PCR assay. This assay, which incorporates six methylation markers, is a part of a multi‐locus blood test known as ColonAiQ, which is used for CRC postoperative surveillance [34]. The efficacy of this test was further corroborated in a comprehensive multi‐center prospective longitudinal cohort study, demonstrating its ability in the early detection of recurrence [35]. Similarly, Wang and colleagues created a model based on five methylation markers to detect MRD for risk assessment of recurrence [36]. In another significant study, Zhu and colleagues amalgamated cfDNA methylation markers with carcinoembryonic antigen, resulting in an MDM score that could be used for recurrence monitoring [37]. Additionally, Jin pioneered a single‐tube methylation‐specific quantitative PCR analysis method, distinguished by its use of 10 different methylation markers. Remarkably, this method enables quantitative analysis of plasma samples containing as little as 0.05% tumor DNA [38].

## 3 The Clinical Applications of ctDNA MRD

**FIGURE 2:**
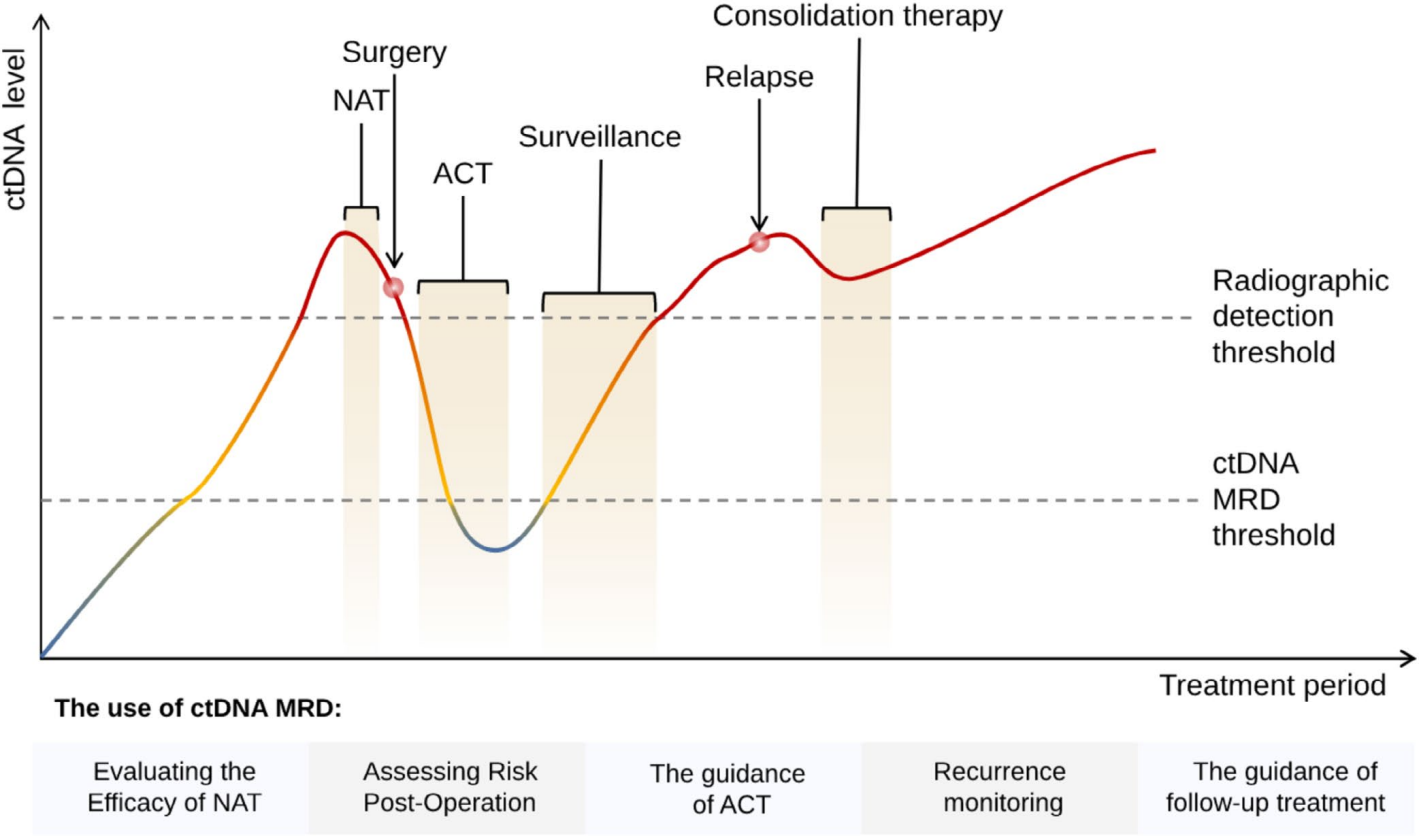
The clinical implications of ctDNA MRD testing during the treatment phase for CRC patients are profound. The figure is adapted from Dasari et al. [39] ctDNA MRD testing proves to be significantly more sensitive compared with traditional methods. After the initial NAT treatment, MRD testing can be employed to assess its effectiveness. Post‐surgery, this testing could serve as a tool to monitor for potential recurrence, and to ascertain whether a patient might benefit from supplementary therapy to minimize the risk of relapse. During surveillance, MRD testing could be utilized to track patient response over time and guide decisions regarding the continuation or alteration of therapy. ACT, adjuvant chemotherapy; NAT, neoadjuvant therapy.

ctDNA MRD monitoring plays a pivotal role in multiple facets of CRC management (Figure 2). First, it facilitates the early detection of recurrence before it becomes clinically observable or identifiable through traditional imaging methods. This early detection allows for immediate intervention, potentially enhancing patient outcomes. Second, ctDNA MRD provides real‐time information, enabling dynamic monitoring of disease progression or response to treatment. This up‐to‐date information allows for swift alterations to treatment plans in accordance with each patient's unique response. Moreover, ctDNA MRD assists in making personalized treatment decisions. It aids in identifying patients with a higher risk of recurrence or progression, guiding the choice of supplementary therapies or the intensity of surveillance strategies. Additionally, ctDNA MRD has demonstrated prognostic value in CRC. In this section, we will delve into the clinical applications of ctDNA MRD during different treatment phases (Table 1).

**TABLE 1:** The representative publications for the clinical relevance of MRD at different stages of CRC.

Author, year	Journal	Patient no.	Stage	Detection method	HR	Evidence
Liu, Wenyang, et al. 2022 [40]	EBioMedicine	60	LARC	Personalized tumor‐informed assay	27.38	Positive MRD was significantly associated with an increased risk of recurrence (HR = 27.38; log‐rank *p* < 0.0001) after NAT.
Wang, Yaqi, et al. 2021 [41]	PLoS medicine	119	LARC	Tumor‐agnostic assay	90.29	Patients who had driver mutations and positive high‐risk features had the highest risk of recurrence (HR = 90.29; *p* < 0.001) after surgery.
Tie, Jeanne, et al. 2019 [42]	Gut	159	LARC	Safe‐sequencing system tumor‐informed personalized ctDNA assay	13	For patients who tested positive for ctDNA after chemotherapy and radiotherapy (HR = 6.6; *p* < 0.001) or after surgery (HR = 13.0, *p* < 0.001), the DFS was significantly worse.
Zhou, Jiaolin, et al. 2021 [43]	Clinical cancer research	106	LARC	Fixed tumor‐informed assay	\	The presence of ctDNA at all four time points (baseline, during nCRT, pre‐surgery, and post‐surgery) was associated with a shorter MFS (*p* < 0.05).
Murahashi, Satoshi, et al. 2020 [44]	British journal of cancer	85	LARC	Tumor‐agnostic assay	\	Changes in ctDNA were an independent predictor of complete response to preoperative therapy in patients with LARC (*p* = 0.0276).
Tie, Jeanne, et al. 2016 [45]	Science translational medicine	230	Stage II colon cancer	Safe‐sequencing system tumor‐informed personalized ctDNA assay	18	In patients who did not receive adjuvant chemotherapy, ctDNA positivity was associated with a higher risk of RFS (HR = 18; *p* < 0.001). In patients who received chemotherapy, the presence of ctDNA after completion of chemotherapy was also associated with poorer RFS (HR = 11; *p* = 0.001).
Chen, Gong, et al. 2021 [46]	Journal of hematology & oncology	240	Stage II/III CRC	Fixed tumor‐informed assay	10.98	After surgery, ctDNA positivity is associated with a significantly higher risk of recurrence (HR = 10.98; *p* < 0.001).
Taieb, Julien, et al. 2021 [47]	Clinical cancer research	2010	Stage III colon cancer	Droplet digital PCR	1.55	ctDNA was an independent prognostic marker for DFS (adjusted HR = 1.55; *p* = 0.006) and OS (HR = 1.65; *p* = 0.011).
Henriksen, Tenna Vesterman, et al. 2022 [48]	Clinical cancer research	168	Stage III CRC	Personalized tumor‐informed assay	7	ctDNA was a strong predictor of recurrence after surgery (HR = 7.0; *p* < 0.001) and ACT (HR = 50.76; *p* < 0.001).
Reinert, Thomas, et al. 2019 [49]	JAMA oncology	130	Stages I to III CRC	Personalized tumor‐informed assay	7.2	After surgery, ctDNA‐positive patients were more likely to recur than ctDNA‐negative patients (HR = 7.2; *p* < 0.001).
Tie, Jeanne, et al. 2019 [50]	JAMA oncology	96	Stage III Colon Cancer	Safe‐sequencing system tumor‐informed personalized ctDNA assay	3.8	ctDNA is associated with worse DFS (HR = 3.8; *p* < 0.001).
Wang, Yuxuan, et al. 2019 [51]	JAMA oncology	58	Stage I to III CRC	Safe‐SeqS	\	In patients who tested positive for ctDNA, the recurrence rate was 77%, while none of the patients who tested negative for ctDNA experienced a recurrence.
Kotani, Daisuke, et al. 2023 [28]	Nature medicine	1039	Stage II–IV resectable CRC	Personalized tumor‐informed assay	10	Postoperative ctDNA positivity is associated with a higher risk of recurrence (HR 10.0; *p* < 0.0001) and is the most significant prognostic factor related to the risk of recurrence in patients with stage II or III CRC (HR 10.82; *p* < 0.001).
Tie, Jeanne, et al. 2022 [29]	New England journal of medicine	455	Stage II Colon Cancer	Safe‐sequencing system tumor‐informed personalized ctDNA assay	\	In the ctDNA‐guided group, the proportion of patients receiving ACT was lower than that in the standard management group (15% vs. 28%). Based on the 2‐year recurrence‐free survival rate, ctDNA‐guided management is not inferior to standard management.
Loupakis, Fotios, et al. 2021 [52]	JCO precision oncology	112	mCRC undergone metastatic resection with curative intent	Personalized tumor‐informed assay	5.8	In 54.4% of patients, postoperative MRD positivity was detected, and by the data cutoff, 96.7% of these patients had experienced disease progression (HR = 5.8; *p* < 0.001).
Pellini, Bruna, et al. 2021 [53]	JCO precision oncology	24	Oligo‐metastatic CRC	Tumor‐agnostic assay	\	The tumor‐agnostic plasma ctDNA analysis identified MRD with a median level of 0.62%, achieving a sensitivity of 95% and a specificity of 100%.

*Note:* The symbol ‘\’ indicates the absence of relevant information.

Abbreviations: ACT, adjuvant chemotherapy; CI, confidence interval; ctDNA, circulating tumor DNA; DFS, disease‐free survival; HR, hazard ratio; LARC, locally advanced rectal cancer; MFS, metastasis‐free survival; NAT, neoadjuvant therapy; nCRT, neoadjuvant chemoradiation; OS, overall survival; RFS, recurrence‐free survival.

We searched PubMed, Embase, and Google Scholar for studies on ctDNA‐based MRD detection in CRC with a search deadline of January 20, 2024. In addition, we searched for references in the included studies to prevent omissions. Inclusion criteria were as follows: (a) patients were diagnosed with colon cancer, rectal cancer, or CRC; (b) investigation of the role of ctDNA‐based MRD detection in the prediction of pCR, tumor recurrence risk, and prognosis, and the guidance of ACT and surveillance; and (c) studies that provided enough information about the ctDNA‐based MRD detection methods. The exclusion criteria were as follows: (a) reviews, letters, and case reports; (b) studies involving comorbidities with other malignant diseases. The study search strategy used a combination of subject terms and free words, and the search strategy was as follows: #1: Circulating Tumor DNA OR ctDNA; #2: Minimal Residual Disease OR MRD OR Molecular Residual Disease OR Measurable Residual Disease; #3: Rectal cancer OR Colon cancer OR Colorectal cancer; #4: Pathological complete response OR pCR OR Prognosis OR Recurrence OR Surveillance; #5: #1, #2, #3, and #4. It is necessary to clarify that the target of MRD detection here is ctDNA somatic mutations. Because the detection of them is mainstream and mature, their application in clinical research is more widespread. However, the technology for methylation detection is still in its infancy, and research is relatively limited, which we have already discussed in the methodology section.

### 3.1 Evaluating the Efficacy of Neoadjuvant Therapy

In managing locally advanced rectal cancer (LARC), the conventional treatment regimen typically involves neoadjuvant chemoradiation (nCRT), followed by a total mesorectal excision (TME) and ACT. However, recent years have seen the rapid development of total neoadjuvant therapy (TNT). This treatment approach shifts ACT and chemoradiotherapy (CRT) to the neoadjuvant phase and is quickly becoming recognized as a standard option in national guidelines.

One of the valuable tools in assessing treatment response to neoadjuvant therapy (NAT) is ctDNA MRD monitoring. By examining the levels of ctDNA in the blood before and during treatment, we can assess the therapy's potency in reducing tumor burden. A decline in ctDNA levels signifies a positive response to treatment, while persistent or increasing levels could indicate resistance or insufficient response [35, 54]. However, Current studies have reported conflicting data regarding the ability of ctDNA status to predict pathological complete response (pCR) during or after CRT. Most studies have actually failed to demonstrate a significant association between ctDNA status and pCR [55]. But research has shown a tendency that a decrease or eradication of ctDNA during treatment correlates with a higher likelihood of achieving a pCR. Conversely, persistent ctDNA could denote residual disease and a lesser likelihood of achieving pCR [40].

In a prospective multi‐center study involving 106 patients with LARC treated with nCRT followed by surgery, it was revealed that ctDNA is an accurate real‐time monitoring indicator. It effectively reflects the tumor burden and the impact of nCRT through serial ctDNA targeted sequencing. Furthermore, all patients with pCR showed no detectable ctDNA MRD [43]. Tie et al. (2019) reported that detectable ctDNA MRD is significantly associated with a worse recurrence‐free survival rate for LARC patients in stage II/III treated with nCRT [42]. Consequently, ctDNA MRD monitoring can assist in personalizing treatment strategies for patients undergoing NAT. Patients demonstrating a good response are marked by a significant decrease or clearance of ctDNA. On the other hand, patients with persistent ctDNA or poor response may necessitate additional or alternative treatment approaches. Moreover, ctDNA MRD monitoring holds prognostic value in assessing the long‐term outcomes of NAT. Studies have indicated that the persistence of ctDNA following NAT aligns with a higher risk of disease recurrence and a poorer prognosis [41, 44]. Such information can be instrumental in risk stratification and guiding post‐treatment surveillance strategies.

### 3.2 Evaluating the Risk of Postoperative Recurrence

Surgery stands as the primary curative treatment for patients with localized, locally advanced, or oligometastatic CRC. However, the looming potential of postoperative recurrence presents a significant issue. The utilization of ctDNA MRD assessment can aid in gauging the risk of this recurrence. On one hand, the monitoring of ctDNA MRD allows for the early detection of residual disease, providing the opportunity for immediate intervention, which can potentially enhance patient outcomes. On the other hand, ctDNA MRD monitoring is predictive and valuable in forecasting the risk of disease recurrence post‐surgery. Postoperative ctDNA level monitoring can assist in risk stratification, thereby informing treatment decisions.

Numerous studies indicate that the persistence of ctDNA MRD following surgery is linked with an increased risk of recurrence and a poorer prognosis [45, 46, 47, 48]. In a study involving a cohort of 130 patients with stages I to III CRC, researchers discovered that ctDNA status was independently correlated with relapse. Moreover, regular ctDNA MRD monitoring unveiled disease recurrence, on average, 8.7 months ahead of radiologic imaging [49]. In another study comprised of 100 stage III colon cancer patients, Tie et al. (2019) found that the detectable ctDNA MRD in post‐surgical samples corresponded with inferior recurrence‐free survival. This suggests that ctDNA MRD after surgery may serve as a promising prognostic marker in stage III colon cancer [50]. Wang et al. (2019) compared the ctDNA MRD with traditional CT and CEA methods in recurrence surveillance. The team found that ctDNA MRD outperformed the other two strategies by a median of 3 months [51]. In the extensive observational GALAXY trial, which analyzed the post‐surgical ctDNA MRD status of 1039 stage II–IV resectable CRC patients, it was found that a positive MRD was linked with a higher recurrence risk. Moreover, it was the most significant prognostic factor associated with recurrence risk in patients with stage II or III CRC [28].

### 3.3 The Guidance of ACT and Follow‐Up Treatment

ACT and follow‐up treatment guidance can be significantly improved with the use of ctDNA MRD monitoring. This method enables the identification of patients who are at an elevated risk of disease recurrence following surgery. By detecting the presence of MRD, those who may derive substantial benefits from more intensive ACT can be pinpointed. Concurrently, patients who test negative for MRD may have the intensity of their ACT treatment reduced. This personalized approach optimizes treatment decisions and holds the potential to enhance patient outcomes.

The comprehensive GALAXY trial reported that stage II or III CRC patients who tested positive for ctDNA MRD after surgery could benefit from ACT [28]. In another randomized intervention trial conducted on stage II colon cancer patients, those in the experimental group who tested negative for ctDNA MRD were able to reduce their ACT treatment. Despite this reduction, no significant difference was observed in 2‐year recurrence‐free survival rates when compared to the control group (93.5% vs. 92.4%) [29]. ctDNA MRD monitoring provides a real‐time evaluation of the patient's response to ACT. By tracking ctDNA levels over time, the dynamics of the MRD can be assessed. A significant decrease or complete clearance of ctDNA may signify a favorable treatment response, potentially indicating that the planned duration of chemotherapy can be adhered to. However, persistent ctDNA MRD may signify the need for an extended treatment duration to further mitigate the risk of disease recurrence. ctDNA MRD monitoring also carries prognostic value for CRC patients undergoing ACT. Various studies have demonstrated that persistent ctDNA MRD during ACT is linked to a higher risk of disease recurrence and a poorer prognosis [48, 56]. This monitoring can assist in risk stratification and guide treatment decisions, such as the intensity of ACT or the consideration of additional therapies.

Notably, ctDNA MRD monitoring can also aid in optimizing the surveillance strategy for postoperative follow‐up [57]. By tracking ctDNA levels, patients at a higher risk of recurrence who may require more frequent or intensive surveillance can be identified. Conversely, patients with undetectable or decreasing ctDNA levels may be suitable candidates for less frequent surveillance, thereby reducing unnecessary testing and healthcare costs.

### 3.4 Monitoring Tumor Load and Response in Metastatic Colorectal Cancer Following Curative Intent Resection

Approximately 50% of CRC patients develop metastatic lesions either at the time of diagnosis or afterward, with the liver being the most common site for metastasis. Surgery, often combined with systemic treatment, is a viable option for about half of these metastatic patients. Monitoring tumor load and response can contribute to more personalized treatment strategies. For instance, it can direct the administration of adjuvant therapy in cases where residual lesions persist post‐resection. It can also help identify when to choose drug holidays during first‐line therapy.

In a preliminary study, researchers investigated the clinical utility of ctDNA MRD for making personalized treatment decisions in metastatic colorectal cancer (mCRC) patients undergoing surgical removal of metastases. A 15‐gene tumor‐informed fixed‐panel assay was used in the process. ctDNA MRD was detected in 0% of patients who experienced a partial pathologic response and 75% of those who had no pathologic response. Furthermore, ctDNA MRD was found in 0% and 80% of patients who underwent complete and incomplete resection, respectively. These findings suggest that ctDNA MRD can potentially monitor chemotherapy response and predict the completeness of surgery in mCRC patients [58]. As part of the PREDATOR clinical trial, Loupakis and colleagues conducted a cohort study involving 112 mCRC patients who underwent metastatic resection with a curative intent. Their MRD status was analyzed based on ctDNA using a tumor‐informed assay. The post‐surgical MRD‐positive rate was 54.4%, and 96.7% of these MRD‐positive patients progressed during the study period, indicating the promising role of MRD in clinical decision‐making [52]. Another study evaluated the feasibility of applying a tumor‐naive method to plasma and urine for MRD detection in mCRC patients undergoing neoadjuvant chemotherapy. The sensitivity of plasma ctDNA analysis was found to be 94%, although the sensitivity of urine ctDNA analysis dropped to 64% [53].

## 4 Ongoing Clinical Trials

Numerous clinical trials are currently being conducted to investigate the clinical significance of ctDNA MRD in CRC. We will provide an overview of the key clinical trials that are using ctDNA‐based MRD for CRC in the following section.

### 4.1 ctDNA MRD as a Prognostic Indicator in CRC

A critical area of investigation involves the use of ctDNA MRD as a prognostic marker in CRC. These trials aim to establish the correlation between the status of ctDNA MRD after NAT, surgery, ADT, or surveillance and long‐term outcomes such as disease‐free survival or overall survival. Some trials are even exploring whether ctDNA MRD can be used to guide treatment decisions in CRC, including adjusting treatment intensity or duration based on MRD status. The ultimate goal is to improve patient outcomes and minimize unnecessary treatment.

NAT plays a significant role in improving survival rates in rectal cancer patients by controlling tumor growth and minimizing the risk of postoperative recurrence and metastasis. Numerous trials are exploring the potential of ctDNA MRD as a biomarker for personalized NAT. For instance, the phase II trial NCT05239546 is a single‐arm study examining neoadjuvant Dostarlimab in stage II and III deficient mismatch repair (dMMR) colon cancer. In this study, researchers are exploring the rate of major clinical response and non‐operative management after 18 weeks of neoadjuvant Dostarlimab, with ctDNA MRD status serving as a critical criterion. Similarly, trial NCT05732389 aims to study immunotherapy in patients with early dMMR rectal cancer, with ctDNA MRD being one of the biomarkers used to identify patients who have entirely eradicated the primary cancer and regional lymph nodes. This could potentially lead to organ‐sparing medical treatments. Trial NCT05964530 is a randomized controlled clinical trial that compares the oncologic efficacy of radical versus local excision for rectal cancer patients who have clinically complete remission to nCRT therapy. This trial uses ctDNA MRD to accurately select patients with pCR. Moreover, trial NCT05081024 is seeking to establish a ctDNA biomarker to improve organ preservation strategies in rectal cancer patients. Finally, trial NCT05601505 is designed to create a ctDNA MRD‐guided NAT strategy and provide appropriate treatment plans and intensities for patients with different disease stages for LARC.

A trial (NCT03776591) is also underway, which is designed to compare the short‐and long‐term outcomes of open D3 and laparoscopic TME with central vascular ligation right colectomy for right‐sided colon cancer. In this trial, ctDNA MRD is employed as a prognostic and predictive biomarker.

ACT serves to eliminate minuscule cancer cells remaining after surgery, thereby reducing the risk of recurrence and improving patient survival. Multiple trials are currently assessing if ctDNA MRD can be optimized as a biomarker for ACT treatment. The NCT05534087 trial is a prospective, phase 3, randomized clinical study that examines whether the early introduction of intensified chemotherapy enhances the prognosis of stage 2–3 colon cancer patients who remain ctDNA MRD positive after standard ACT, as compared with standard treatment. Conversely, NCT04050345 is a multi‐center, prospective, randomized study aiming to demonstrate that a de‐escalation strategy of ctDNA‐guided ACT is not inferior to standard of care treatment in patients with high‐risk stage II or stage III CRC with negative MRD. The NCT05815082 trial seeks to ascertain if the prognosis of watching and waiting (W&W) is non‐inferior to ADT in postoperative ctDNA MRD‐negative CRLM patients. The CIRCULATE trial (NCT04120701) aims to enhance the care of patients post colon tumor surgery based on ctDNA MRD status. Additionally, the CIRCULATE study (NCT04089631) compares the disease‐free survival in colon cancer UICC stage II patients who are MRD positive postoperatively, with or without capecitabine. The NCT04920032 is a phase 1b, single‐arm, non‐randomized, open‐label clinical trial investigating the efficacy of adjuvant trifluridine and tipiracil (TAS‐102) in combination with irinotecan in patients with ctDNA MRD‐positive colon adenocarcinoma.

Following adjuvant therapy, several trials are in progress to determine whether additional treatment could enhance the prognosis of MRD‐positive patients. For instance, the NCT05350501 trial evaluates the combined use of the microbiome‐derived therapeutic vaccine EO2040 with nivolumab in patients with ctDNA MRD‐positive CRC stage II, III, or IV upon completion of standard curative therapy. The NCT05240950 trial is a phase I clinical study focusing on patients with MRD‐positive status after R0 resection of CRLM, aiming to conduct a preliminary exploration of anti‐CEA CAR‐T cell therapy and assess its safety and efficacy. The NCT05031975 trial investigates the combination of temozolomide and irinotecan as a consolidation non‐cross‐resistant therapy in a ctDNA MRD‐driven interventional trial.

Surveillance is crucial in helping doctors understand the patient's condition and treatment response, thus enabling them to adjust treatment regimens accordingly. The NCT05161585 is an open, prospective, randomized controlled phase II cohort study that explores whether ctDNA MRD can guide a personalized surveillance strategy post‐surgery.

Finally, the NCT05234177 is an observational multi‐center study evaluating the value of longitudinal ctDNA MRD monitoring in addition to its standard‐of‐care therapy and disease surveillance for CRC patients.

### 4.2 ctDNA‐Based MRD Detection Methods for Early Detection of Recurrence

There are currently several ongoing trials assessing the efficacy of ctDNA MRD as an early biomarker for disease recurrence. These studies typically involve post‐treatment monitoring of ctDNA levels to pinpoint patients at an increased risk of recurrence, thereby enabling timely intervention.

Among these trials, the REVEAL study (NCT05674422) is a prospective, multi‐center investigation focusing on the potential role of ctDNA‐based MRD detection methods in predicting relapse in LARC patients treated with TNT, followed by either W&W or TME. Similarly, the CORRECT‐MRD II study (NCT05210283) is enrolling patients who have undergone comprehensive surgical resection for stage II or III CRC. These patients will be monitored for a minimum of 3 years and a maximum of 5 years to track potential recurrence. The NCT05444491 study, on the other hand, employs a dynamic monitoring approach for predicting the prognosis of stage I–IV CRC patients who have received radical surgical resection. This is achieved by detecting MRD based on a polygene methylation strategy. Finally, the POSTCA trial (NCT03737539) is a prospective, multicenter, observational, single‐blinded controlled study. It aims to explore whether the postoperative methylation‐based MRD detection method can predict postoperative recurrence. It also investigates whether dynamic monitoring of postoperative ctDNA methylation could be a more timely indicator of tumor recurrence than traditional imaging examinations.

### 4.3 The Study of ctDNA MRD Methodology

In addition, there are several trials focusing on methodological research. For instance, NCT03868215 is a forward‐looking clinical study that evaluates the sensitivity of plasma ctDNA methylation haplotypes in the detection of MRD. Furthermore, the MiRDA‐C Study (NCT04739072) explores whether ctDNA, along with other tumor‐related molecules like RNA and proteomic alterations sourced from plasma, can aid doctors in predicting the recurrence or metastasis of CRC.

## 5 Prospective and Limitations in Routine Clinical Practice

Although current detection methods for MRD predominantly focus on genomic point mutations and sequence alterations, the field is evolving to incorporate a range of marker combination tests. Examples of these tests include the integration of protein or CNA data. It is anticipated that emerging technologies such as cfDNA fragmentomics, methylomes, or nucleosome imprinting will become increasingly utilized for MRD detection [59]. At present, blood is the favored medium for MRD testing. However, as detection technologies advance, non‐invasive samples such as urine and feces are likely to become more prevalent testing subjects. Feces, in particular, have already proven instrumental in the screening and early diagnosis of CRC [60].

There has been a shift in studies evaluating ctDNA MRD testing from retrospective analysis to prospective studies. The focus of the field is now on interventional, randomized controlled trials. Both randomized and non‐randomized clinical trials assessing the potential of ctDNA MRD guidance for treatment and its prospective clinical applications are currently in progress, marking a significant step forward. The design of ctDNA MRD‐directed clinical trials is being approached in various ways. Some trials are pre‐screening patients using ctDNA MRD tests, subsequently randomizing them to adjuvant treatment groups to guide treatment intensification or moderation. Others evaluate the potential clinical benefits of stratification based on ctDNA MRD by randomizing patients to either intervention or observation groups. Rapidly accumulating evidence in the field supports the establishment of ctDNA MRD‐guided clinical trials, underscoring the value of liquid biopsies in guiding treatment decisions. Despite the challenges that remain in implementing ctDNA MRD monitoring in CRC, significant progress continues to be made.

It is necessary to point out that there are some limitations of the ctDNA MRD test in clinical applications. First, the primary barrier is the need for extensive validation and standardization of ctDNA MRD assays. Variability in assay sensitivity, specificity, and reproducibility across different platforms can hinder their reliability and acceptance. Establishing standardized protocols and guidelines is essential to ensure consistent results across laboratories. Second, demonstrating the clinical utility of ctDNA MRD tests is crucial for their integration into practice. Large‐scale studies that provide evidence on how these tests can guide treatment decisions and improve patient outcomes are needed. Moreover, the incorporation of these tests into clinical guidelines and recommendations by professional societies is necessary to drive their adoption. Third, regulatory approval and reimbursement by healthcare systems are critical for widespread clinical use. The pathway to regulatory approval requires comprehensive clinical evidence, and securing reimbursement requires demonstrating cost‐effectiveness and value to healthcare providers and payers. Fourth, the high costs associated with ctDNA MRD testing, including the necessity for advanced technology and skilled personnel, can be prohibitive. The cost of ctDNA testing can be significant and may not be fully covered by insurance, which can pose a financial burden on patients. This is particularly important in healthcare systems where the cost‐effectiveness of new technologies is a crucial consideration. The high costs may limit accessibility and equitable use of the test across different patient demographics. Reducing costs through technological advancements and increased competition could facilitate broader access. Future studies should focus on cost–benefit analyses and explore ways to reduce costs through technological advancements or policy changes. Besides, receiving a positive ctDNA test result can lead to significant anxiety and stress for patients, especially in cases where there is a delay before clinical or radiographic confirmation of disease. It is important to provide adequate psychological support and counseling to help patients understand the results and manage their anxiety. Healthcare providers should be trained to communicate the implications of ctDNA test results effectively. Finally, there can be a considerable lead time between detecting ctDNA and confirming disease through radiographic evidence. This lead time may vary depending on the type of cancer and its progression. Although early detection via ctDNA can be beneficial, it also necessitates careful clinical management to avoid unnecessary interventions. Ongoing research is needed to better predict which positive ctDNA results will lead to clinical disease, thereby refining the use of ctDNA as a monitoring tool.

## 6 Conclusions

The incorporation of ctDNA MRD surveillance in CRC showcases immense potential. This methodology confers numerous benefits, including the prompt detection of relapse, comprehensive assessment of treatment outcomes, personalization of treatment strategies, and effective prognosis. Without a doubt, this innovation is ushering in a paradigm shift in the field of CRC treatment. However, we need to soberly realize that the adoption of ctDNA‐based MRD testing into mainstream clinical practice faces several challenges.

In conclusion, while ctDNA MRD tests hold great potential for improving cancer management, addressing these barriers through collaborative efforts among researchers, clinicians, regulatory bodies, and industry stakeholders is essential to realize their integration into clinical practice.

## Author Contributions

Shifu Chen, Tianhao Mu, and Ming Liu contributed substantially to the conception and design of the study. Ming Liu drafted and revised the manuscript. Ming Liu, Tianhao Mu, Mingyan Xu, Jia Gu, and Shifu Chen provided critical revision of the manuscript. All authors read and approved the final manuscript.

## Ethics Statement

The authors have nothing to report.

## Consent

The authors have nothing to report.

## Conflicts of Interest

The authors declare no conflicts of interest.

## Data Availability

Data sharing not applicable to this article as no datasets were generated or analysed during the current study.
